# Gene Transmission in the One Health Microbiosphere and the Channels of Antimicrobial Resistance

**DOI:** 10.3389/fmicb.2019.02892

**Published:** 2019-12-17

**Authors:** Fernando Baquero, Teresa M. Coque, José-Luis Martínez, Sonia Aracil-Gisbert, Val F. Lanza

**Affiliations:** ^1^Department of Microbiology, Ramón y Cajal University Hospital, Ramón y Cajal Institute for Health Research (IRYCIS), Madrid, Spain; ^2^Centro Nacional de Biotecnología, Consejo Superior de Investigaciones Científicas (CSIC), Madrid, Spain; ^3^Bioinformatics Unit, Ramón y Cajal University Hospital, Ramón y Cajal Institute for Health Research (IRYCIS), Madrid, Spain; ^4^CIBER in Epidemiology and Public Health (CIBERESP), Madrid, Spain

**Keywords:** one health, accessory genes, resistance genes, gene flow channels, microbiome merging

## Abstract

Antibiotic resistance is a field in which the concept of One Health can best be illustrated. One Health is based on the definition of communication spaces among diverse environments. Antibiotic resistance is encoded by genes, however, these genes are propagated in mobile genetic elements (MGEs), circulating among bacterial species and clones that are integrated into the multiple microbiotas of humans, animals, food, sewage, soil, and water environments, the One Health microbiosphere. The dynamics and evolution of antibiotic resistance depend on the communication networks linking all these ecological, biological, and genetic entities. These communications occur by environmental overlapping and merging, a critical issue in countries with poor sanitation, but also favored by the homogenizing power of globalization. The overwhelming increase in the population of highly uniform food animals has contributed to the parallel increase in the absolute size of their microbiotas, consequently enhancing the possibility of microbiome merging between humans and animals. Microbial communities coalescence might lead to shared microbiomes in which the spread of antibiotic resistance (of human, animal, or environmental origin) is facilitated. Intermicrobiome communication is exerted by shuttle bacterial species (or clones within species) belonging to generalist taxa, able to multiply in the microbiomes of various hosts, including humans, animals, and plants. Their integration into local genetic exchange communities fosters antibiotic resistance gene flow, following the channels of accessory genome exchange among bacterial species. These channels delineate a topology of gene circulation, including dense clusters of species with frequent historical and recent exchanges. The ecological compatibility of these species, sharing the same niches and environments, determines the exchange possibilities. In summary, the fertility of the One Health approach to antibiotic resistance depends on the progress of understanding multihierarchical systems, encompassing communications among environments (macro/microaggregates), among microbiotas (communities), among bacterial species (clones), and communications among MGEs.

## Introduction: One Health as a Communication Space

A recent reformulation of the classic One Health approach emphasize the role of interconnected (and hence geographically close) ecosystems in the emergence and dissemination of traits that influence local human, animal, plant, and integrated environmental health (ecosystem health), such as antibiotic resistance (Knapp et al., 2009; Dandachi et al., 2019; Hernando-Amado et al., 2019; Scott et al., 2019; van Bruggen et al., 2019). In fact, antibiotic resistance has been considered the quintessential One Health issue (Robinson et al., 2016). One Health is an ecological concept, and antibiotic resistance is a trait linked to microbiotas, microbial assemblages that are organized and evolve by fundamental processes of community ecology (Costello et al., 2012). Community ecology is a science of environmental communication. As with any communication process, the success of antibiotic resistance transmission is based on three aspects: the communication space, the vehicle for the communication, and the interpretation by the recipient of the message (Baquero, 2017, 2018).

We can consider three communication spaces in the One Health dimension: (1) communication networks between humans, animals, and plants environments, and also with the external environments; (2) communication networks between microbiomes belonging to the above environments, and their sub-environments; and (3) communication networks between various bacterial species within these microbiomes (resulting from microbiome merging). The edge density (density of interconnecting links) in these networks should be proportional to the possibility of the spread of antibiotic resistance genes in this One Health ensemble. In addition to the communication networks, the elements of transmission are also relevant to defining in depth the process of transmission that, in the case of antibiotic resistance, largely relies on the hierarchical organization of antibiotic resistance elements (Baquero, 2004), which allows a selection space with various levels. Selection is then a critical element for the success of the communication because it provides for interpretation of the transmitted message. Antibiotic-resistant mutants are present in all bacterial populations, and, of course, antibiotic resistance is very ancient in biological times (D’Costa et al., 2011). The current mobile genetic elements (MGEs) carrying antibiotic-resistance genes (as plasmids, transposons, or integrons) were already circulating in Enterobacteriaceae long before the use of antibiotics (Datta and Hughes, 1983; Rowe-Magnus et al., 2001); these elements were rapidly colonized with antibiotic resistance genes, in part evolving from pre-resistance genes, at the time of anthropogenic antibiotic use and selection. However, it is this utilization that provides a meaning to antibiotic resistance, which allows for communication and hence the spread of the message, in this case antibiotic resistance.

## Communication Between Humans, Animals, Plants, and Local External Environments

Communication is proportional to the density and connectivity (capacity for interconnection) of such entities. The coincidence of dense human populations with a high density of terrestrial vertebrate animals (those with a higher probability of microbiome merging), both sharing a common environment, provides a strong opportunity for frequent biological interactions, particularly microbiome **merging** (Ley et al., 2008). Frequent interactions between human (and pre-human) and other animal microbiomes started by hunting and scavenging meat activities, but were significantly increased during the Neolithic period, with the invention of farming and the associated increase in the size of human populations stably coexisting with animals in the same habitat (Armelagos et al., 2005; Fournie et al., 2017; Roughgarden et al., 2018). However, this interaction has greatly increased in the last century, with sociodemographic changes in population, dietary habits, particularly the increase in animal production and meat consumption in low and middle income countries, and the green revolution in agriculture (Tilman, 1998; Tilman et al., 2011; Van Boeckel et al., 2019).

### Communication and Population Sizes

Agriculture currently uses 11% of the world’s land surface for crop production. Since 1961, while total cultivated land has shown a net increase of 12 percent to 2009, land under irrigation has more than doubled (FAOSTAT)^1^. Farming activity has escalated since World War I to reach massive proportions. The world cattle inventory in 2018 is at one billion heads, with half of these animals in India and Brazil, and the third-most in China^2^. Data from the Food and Agriculture Organization of the United Nations indicates that the world’s average stock of chickens is estimated at almost 23 billion, and pigs account for 770 million (Lawrence, 2019; Metcalfe, 2019). Interestingly, such an “animal invasion” has frequently occurred in combination with a decline in animal diversity due to anthropogenic selection of a limited range of animal varieties of economic interest. The predictable effect is the increased possibility of interactions among large numbers of a few animal types with large numbers of humans.

There has also been a spectacular increase in the population of particular crop plants due to the technologically driven “Green Revolution” starting in 1950s and 1960s (Matson et al., 1997), which has recently intensified by intercropping, growing two or more crops in proximity (Martin-Guay et al., 2018). We should not forget frequent animal co-culturing (interbreeding), or animal and plants co-production. The net result is the enlargement of fields promoting a mixing ecology of vegetables, animals and humans. For instance, intensively managed rice farming paddy soils might constitute unique agroecosystems, providing opportunities for mixing different microbiomes (Tanskul et al., 1998). As we will state in the next section, the dimensions of this mix are and will be critical in shaping the problem of antibiotic resistance.

### Connectivity Facilitates Microbiome Merging and Hybridization

**FIGURE 1:**
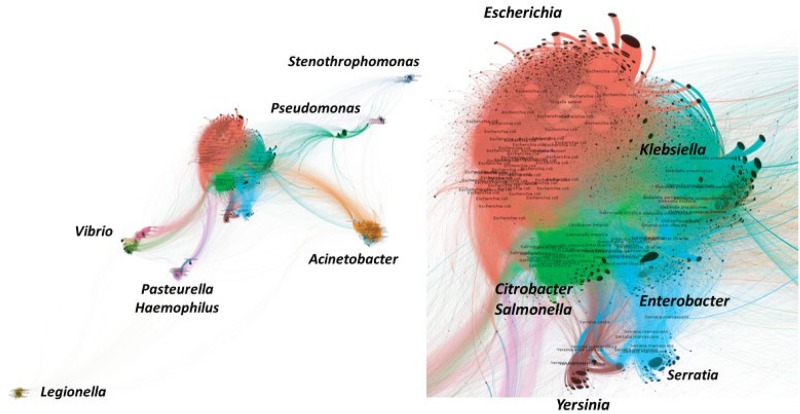
Bipartite network illustrating the accessory genes (proteins) gene flow among species of the major taxons of Gamma-Proteobacteria. Connections between bacterial species indicates that the same accessory gene is shared by both. The distance between species (genus, in italics) is proportional to the number of connections. On the right, detail of the “core” of *Enterobacteriaceae* species sharing accessory genes. Flow of antibiotic resistance genes should correspond to the flow of accessory genes. “Trumpet-like” patterns on the surface of some clusters correspond to accessory genes that are unique for a particular strain (not connected with any other). Reproduced with permission from Lanza et al. (2018).

However, the density of contacts also depends on anthropic local interventions, which are deeply influenced by sociology and economics. Urban ecosystems have established barriers to exclude contact with rural (farming) areas dominated by animals and plants. Such barriers are still weak in some developing countries; thus, the number of interactions is high. However, the challenge of feeding 11 billion people in 2050 implies an increase in contact between humans and animals in the agricultural use of water, antibiotics, and fertilizers, all of which are risk factors for the development of infectious diseases and antibiotic resistance (Rohr et al., 2019). In summary, if some general trends occur at a global scale, such as the increase in the population of humans, food animals, and plants, the investigation of local conditions influencing One Health dynamics will become essential to shaping the dimension of the local risk of interactions based on **microbiome merging** (Ley et al., 2008; Pehrsson et al., 2016; Flandroy et al., 2018; Trinh et al., 2018). Note that microbiome merging is expected to occur preferentially among hosts sharing some basic common features. For instance, the intestinal microbiota of most vertebrates is dominated (in various proportions) by the same taxa, typically Firmicutes, Bacteroidetes, and Proteobacteria, regardless of whether the host is herbivorous or omnivorous, including marine mammals; a quite different pattern is obtained in invertebrates (Ley et al., 2008; Nelson et al., 2015; Colston and Jackson, 2016). A more detailed work is waited for lower taxa, more dependent on the habitat conditions. However, at the level of species, the intestine-adapted microorganism *Escherichia coli* is ubiquitous in mammals (Gordon and FitzGibbon, 1999). In fact, the coincidence of taxa reflects the fact that evolution of animals has occurred in parallel to the evolution of their microbiomes (McFall-Ngai et al., 2013). The terms “phylosymbiosis” was in fact coined to refer to the concordance between a host phylogeny and microbial community (microbiome) dendrogram (Theis et al., 2016). Horizontal accessory gene transfer, and the potential spread of antibiotic resistance genes, occurs preferentially among phylogenetically related bacteria (as is illustrated in Figure 1), even though transfer might bridge different taxonomic levels (Boto, 2009).

It is also worth mentioning that most probably one of the first barriers to preventing colonization by bacteria originated in other hosts (eventually antibiotic resistant) is the presence of a dissimilar microbiome able to outcompete the alien novel microorganisms. Gut microecology is based on ensembles of bacteria that have evolved in protocooperation or synergy; if the “right partners” are absent, alien organisms are difficult to establish, or might produce dysbiosis and reduction in diversity. For instance, because of the influence of pig’s microbiota, the microbiota of farm workers is less diverse than in neighbor villagers (Sun et al., 2017). Such reduction in microbial diversity decreases colonization resistance (Van der Waaij et al., 1971; Buffie and Pamer, 2013) facilitating acquisition of antibiotic resistant strains. That has a correspondence in the host-occupied environments, as it was observed in built environments (as private homes, workplaces, hospitals) where a loss of microbiomes’ diversity following interventions to decrease microbial load correlates with an increase of antibiotic resistance (Mahnert et al., 2019).

## Communication Between Microbiomes of Different Species

### Microbiome Communication in Virgin and Stressed Habitats

The increasing density and connectivity of individuals of different species (humans, animals, plants) produces a major increase in the total size of a limited number of particular microbiomes, facilitating microbiome merging, a condition for the interbacterial spread of antibiotic resistance genes (Hernando-Amado et al., 2019; van Bruggen et al., 2019). This effect is possibly modified by the **short lifespan of farm animals**, reaching their slaughter age much earlier than their potential maximum life expectancy, approximately 10 times earlier for cattle, 50 for pigs, and 100 times for chickens^3^. On one hand, the slaughtering process eliminates the microbiota, including antibiotic-resistant populations (except in the case of slaughterhouse wastewater contamination). On the other hand, the replacement with newborn animals implies an intensive buildup of new microbiotas (high rate of microbiota reproduction). During the construction of the microbiota in each new individual, not only the access of microorganisms from other hosts and environments is facilitated, but the higher bacterial (and community) replication rate in a underexploited nutrient-rich habitat provides more opportunities for genetic exchange, and if antibiotics are present in the environment, selection of resulting resistant populations.

The emergence of novel opportunistic pathogens of non-human environmental origin, particularly among particular species or clones in Enterobacterales, *Acinetobacter*, *Pseudomonas*, and *Enterococcus* associated with the use of antimicrobial agents, might be linked to this process. When involved in clinical infections, these organisms necessarily colonize the mucosal surface of the host and interact with the local microbiota, most probably with phylogenetically close organisms sharing similar Hutchinsonian niches. An Hutchinsonian niche an imaginary space with many dimensions (hypervolume), in which each dimension or axis represents the range of some environmental condition or resource required for the optimal growth of a sublineage or genotypic group (Holt, 2009).

Environments under stress (natural or anthropogenic, including antimicrobial agents) tend to reflect reduced microbial alpha diversity, the number of species/clones harbored in particular microbiota (Rocca et al., 2018). To a certain extent, stress tends to produce species-deficient habitats, providing the opportunity for alien colonizers to invade. Stress-driven reduction of species and clones reduces the diversity of microniches that are dependent on their functions. Empty niches (in terms of resources that are not exploited) act as attractors for members of neighboring organisms. Many of these free-microbe-specific microniches are now occupied by more stress-resistant, less niche-specific organisms, which originated from other microbiotas. As in the case of the naturally bacterial-free intestinal habitat of newborns, the opportunity arises for microbiome collisions and hybridizations. Reduction in alpha diversity could be expected to reduce the possibility of asymmetric dispersion of a variety of rare organisms in neighboring habitats (reduction in beta diversity). However, the collision of microbiotas creates new configurations in which rare taxa could emerge (Rocca et al., 2018, 2019). The application of zeta diversity metrics (measuring the degree of overlap in the type of taxa present between a set microbiotas) will be most useful to illustrate the spatial structure of multispecies distributions in various environments, and therefore the dimensions of microbiome merging (Hui and McGeoch, 2014).

### The “Shared Microbiome” in the Communities of Hosts

Certainly, the case of mother-child microbiome transmission illustrates the importance of shared microbiomes among closely related families of hosts (Arrieta et al., 2014). In fact, this is a case of “microbial community reproduction,” given that not only individual cells, but communities such as microbiotas also reproduce and evolve (Baquero, 2014; Gordo, 2019). As in other cases of biological reproduction, the reproducibility of the original microbiota pattern is imperfect, that is, not maintained in its integrity; thus, differences can be detected among individuals (Vallès et al., 2014). Most importantly, the reproduction of a recently acquired microbiota among closely related newborns maintains a highly conserved composition; with time, however, “microbial evolution within hosts” both in terms of species genetic evolution and migration of strains, takes place (Theis et al., 2016), increasing differences between them, which is beta diversity (Garud et al., 2019; Gordo, 2019). Thus, animals that are slaughtered early should have a more homogeneous microbiota, and the amount of this particular type of microbiota should increase in the environment.

In a shared environment, the dispersion of microbiota in highly related hosts, for instance in the same or closely related species and under similar dietary regimens (Moeller et al., 2013; David et al., 2014) ensures that the more advantageous variants emerging in a particular individual (Garud et al., 2019) can be spread to the other members of the host’s community. To a certain extent, there is a “collective microbiota optimization” favoring the health of the herd. This phenomenon is the other side of the coin of that giving rise to deleterious epidemics. Indeed, microbiota homogenization in ensembles of hosts is a major factor facilitating specific interactions and genetic transfer.

### Microbiota Community Coalescence

The process of microbiome merging and hybridization that might give rise to (at least partially) novel assemblies of bacteria originated in different environments is a phenomenon known in ecology as “**community coalescence**” (Rillig et al., 2015; Rillig, 2017). The combined increase in number and collapse in diversity of animals interacting with humans should facilitate reiterative coalescence events between the same microbiotic types, and thus interbacterial gene transfer.

The dynamics of microbiome merging are insufficiently understood (Roughgarden et al., 2018). New observations, suggesting the **modular structure of microbiota** (Earle et al., 2015; Tropini et al., 2017), indicate the possibility of a “**recombinational merging**” within and between microbiomes, eventually resulting in emerging taxa and emerging communities (Rocca et al., 2018, 2019). In the soil, specific microbial aggregate communities can be considered “microbial villages,” periodically connected through wetting events, where soil moisture is increased as a result of rainfall infiltration, allowing for the transfer of bacterial organisms and genetic material (Wilpiszeski et al., 2019).

Fecal transplantation provides an excellent case study for microbiota coalescence, at least in closely related types of hosts. In fact, bacterial species that might graft in the receptor host could probably be predicted by the abundance and phylogeny of bacteria in the donor and the pre-transplant receptor (Smillie et al., 2018). In the immediate period after transplantation, the invading microbiota from the donor tends to prevail, however, the before-transplant microbiota tends to progressively be restored, typically after 3 months (Seekatz et al., 2014). This is a type of “resilience effect,” where the recovery of the remaining minorities of substituted populations, or the reacquisition of the lost strains, or their functional equivalents from the environment, reconstruct the original pattern of the microbiota (Allison and Martiny, 2008). However, novel strains or species introduced by transplantation procedures can colonize without necessarily replacing the indigenous strains or species of the recipient; in fact, the presence of a kin-strain might facilitate colonization (Li et al., 2016). Thus, for a prolonged period of time there is a transient hybrid microbiota in which new consortia can be established, facilitating genetic interactions.

### Generalist Bacterial Shuttles

Intermicrobiome communication can be facilitated by shuttle bacterial species (or clones within species) belonging to generalist taxa, able to multiply in the microbiomes of various hosts. In fact, a part of these taxa can be considered “colonizing opportunistic pathogens” (Price et al., 2017). The specialist-generalist paradigm predicts that specialists will have local advantages (narrow resource utilization but high performance), and should predominate in specific microbiotas; whereas generalists, which are probably less abundant locally (broader resource utilization but lower performance), are able to colonize diverse microbiotas (Mariadassou et al., 2015). Generalist taxa are identified by a wide Levin’s niche width index, detecting a broad range of niche conditions that a species could inhabit and successfully survive and reproduce, which can be obtained considering the proportion of operational taxonomic units (reflecting the bacterial diversity) in various microbiomes (Pandit et al., 2009). These taxa act as “microbial hubs” in scale-free networks, linking diverse microbiotas. Eventually, these shuttle taxa could have a deeper effect on the architecture of the recipient microbiota than could be expected by their abundance (Muller et al., 2018), thus representing “**keystone species**” (whose presence facilitate the establishment of many others) such that these species disappear or have reduced fitness, and the health (optimal composition) of several apparently unrelated microbiomes might be impaired (Berry and Widder, 2014).

### Niche Overlap and Metabolic Landscapes

Incoming bacteria might compete for those organisms in the recipient microbiota sharing the same function (functional redundancy) (Koskella et al., 2017). The complexity of most natural habitats likely frequently precludes the extinction of competing organisms, following a kind of “imperfect exclusion principle.” Indeed, microbiome merging depends on the local metabolic landscape, which largely determines the “Hutchinsonian niche” of bacterial species and communities (Holt, 2009).

Certainly, the degree of microbiome merging could be positively influenced by bacterial species **niche overlap**, which occurs when incoming and recipient organisms share the same resources and other ecological factors (Cornell, 2012; Moeller et al., 2013). Local microecological similarities between some areas (habitats) in the colonized hosts or between hosts and external environments should facilitate merging. However, available data regarding the microecology of colonizable habitats, such as the intestine of humans and animals, remain scarce (Baquero, 2015). Progress in metabolomics and metabolic reconstruction will soon remediate this important gap (Abubucker et al., 2012). In fact, the structure of microbiomes is ecologically determined by their metabolic networks (Muller et al., 2018). The field termed “metabobiomics” has been suggested to study the correlations between the composition of the intestinal microbiome and the metabolome (Xu et al., 2015*).*

### Ecological Microbiota Mixing in Gradients

An important but insufficiently explored issue is the role of ecological gradients, containing a series of partially overlapping niches, in the bridging process of microbiome merging. For instance, members belonging to different host microbiomes (including animals, plants, soils, humans) might transiently coexist in inland water sewage, wastewater treatment plants, in contaminated agricultural puddled areas, or simply in the soil of farms and human habitats in regions with poor sanitation (Baquero et al., 2008; Berendonk et al., 2015; Pärnänen et al., 2019). Transient, but reiterated coexistence between various microbiotas (or microbiotic modules) might provide the opportunity for new associations of bacteria originated in different hosts, led by generalist taxa. In areas with poor sanitation, such associations could be introduced by continuous exposure to contaminated water or food in the microbiota of humans or animals, providing the opportunity for microbiome evolution, eventually reaching a generalist-like, “broader spectrum microbiota” (Fondi et al., 2016).

## Communication Between Bacterial Species: Mobility of Antibiotic Resistance Genes

### Connectivity of Microbial Genetic Networks

Ecological connectivity is certainly the basis of the formation of gene exchange communities, as well as of common mechanisms of niche construction or even of task distribution (Smillie et al., 2011; Fondi et al., 2016). However, gene exchange might favor the members of the community in an asymmetrical manner, particularly if the transmitted element is of great profit, as occurs in the case of antibiotic resistance. This asymmetry could have consequences for increasing the population sizes of the organism under selection, and thus for connectivity with other populations. In this regard, it would be important to document whether the recent spread of MGEs carrying antibiotic resistance genes across various microbiotas is contributing to remodeling (maybe expansion) of the borders of gene exchange communities, by recruiting novel partners able to communicate with new potential gene receptors. This possibility is in line with the concept of cumulative genetic evolution or “genetic capitalism,” in which the more adapted organisms increase in population size and consequently in connectivity and genetic interactions (Baquero, 2004).

One aspect that is not yet fully understood is the construction of gene exchange communities, particularly when the microbiome emerges *de novo* in virgin habitats, as in newborns (Skippington and Ragan, 2011; Boon et al., 2014; Mansfeldt et al., 2019). An important aspect to be considered is whether these communities “replicate” in newborns, maintaining an identical member composition, or whether new members (originated in other hosts or environments) are accepted in the genetic exchange club in these early stages of microbiota construction. A model based on puzzle construction (Baquero and Nombela, 2012) suggests that the building of the microbiota depends (as in the pieces of a puzzle) on the successive mutual recognition of the components of the community, which is independent from the order of accession. However, this is a “degenerated puzzle,” so that various pieces (different but functionally related species) can occupy the same space and establish the same, or very similar, interactions with the other pieces. This situation provides an opportunity to create variant genetic exchange communities.

Under natural circumstances, transenvironmental colonizers are probably a minority among those that are transferred. However, exposure to high inocula and/or population amplification by antibiotic selection can facilitate local adaptation, successful colonization, and integration into new genetic exchange communities (Baquero, 2018; Sheppard et al., 2018).

Communication between microbiomes is a condition for the propagation of genes between bacterial populations. Let us first clarify that the concept of antibiotic resistance genes is extremely anthropocentric. With few exceptions, possibly antibiotic producers (Davies, 1990), antibiotic resistance genes were not born to resist antibiotics (Linares et al., 2006; Martínez, 2012). They simply belong to a large pool of genes encoding paraphysiological adaptive functions. In this section, we will focus on trans-specific mobile antibiotic resistance genes: those that can be detected, with a high degree of nucleotide identity, in various bacterial species.

### The Mobile Accessory Genome

The pangenome is the gene repertoire of a given bacterial species, that is, the ensemble of all genes contained in all individuals within the species (Tettelin et al., 2008). In these studies, the notion of “species” should be defined in robust way, the ensemble of organisms with at least 95% of average nucleotide identity, as obtained in all-versus-all sequences comparisons. In most species, the pangenome is much larger than the “core genome,” accounting for genes that are contained in every individual of the species, involved in the basic machinery of cell functioning. The difference between the pangenome and the core genome is due to a collection of genes that can be present or not in a given population inside the species, unique genes responsible for functions that are adaptive, niche-specific, and eventually of a contingent nature. The genes are considered “dispensable” (at least in basic culture conditions) or “accessory” (complementing the core genome). The fact that they are not present in all individuals of a species means that they can be gained or lost; in addition, the same genes (with high sequence homology) can be found across various species, indicating interspecific mobility (Segerman, 2012). Interspecific gene mobility can be neutral if the genes are transported and acquired unspecifically only because they are hosted in MGEs, but do not provide a current benefit for the recipient bacteria. These genes can, however, be “markers” to trace genetic transfer. In many cases, interspecific accessory gene flow has an adaptive function, and the transmitted genes are critical for survival in particular environments and contribute to bacterial eco-specific diversification (Wiedenbeck and Cohan, 2011; Rouli et al., 2015). Of course, acquired antibiotic resistance genes are accessory genes, and are transferred among microorganisms by using the same MGEs than other pre-antibiotic accessory genes encoding inhibitors-resistance, including those determining resistance to heavy metals (van Hoek et al., 2011).

The “**convergence of adaptive needs**” among bacterial species should foster interspecific communication. Antimicrobial exposure is forcing many different organisms to survive, and there are a limited number of genes able to provide resistance. If these genes are carried by the mobilome of a particular microbiota where these species can coexist, an increased possibility of interspecific genetic transfer is expected to occur. This transfer suggests the interesting possibility that antibiotic exposure could trigger interspecific gene flow. Certainly, the accessory genes (constituting most of the pangenome) reflect the ecological needs of organisms and might be useful to redefine species and subspecies (Laing et al., 2010; Caputo et al., 2015).

Species located in very stable, reduced, highly specialized niches are less exposed to the gene-traffic circuit; thus, their pangenome is close to their core genome (Martínez et al., 2017; McInerney et al., 2017). Significant examples are *Listeria monocytogenes*, or *Legionella pneumophila*, able to exploit intracellular (stable, isolated) niches, which have larger core genomes (“closed genomes”) than other members of their phylogenetic relatives, indicating less exposure to horizontal gene transfer (Gomez-Valero et al., 2011; Collins and Higgs, 2012). On the contrary, many bacterial organisms of importance in public health, and particularly those able to colonize different environments, have an “open pangenome” that is open to the immigration (capture) of a wide variety of genes. In the case of *E. coli*, a recent study has estimated a pangenome of 15,950 genes in 60 strains, 13,076 for the accessory genome and 2874 for the core genome (Her and Wu, 2018). In one of the largest available studies of *E. coli* (more than 2000 genomes), the authors estimated 3188 core gene families (defined as being present in 95% of genomes) and approximately 90,000 unique gene families (Land et al., 2015). The discovery of a cumulative number of new genomes in species with “open pangenomes” suggests that the number of potential accessory genes has no real limit (Lapierre and Gogarten, 2009). Why does this massive amount of horizontal genetic flow not cause a significant phylogenetic disruption in bacterial species? Probably because the preservation of the species’ “core genome” in different circumstances and environments is assured by the acquired accessory genome (Ochman et al., 2005). On the other hand, in most cases, the recent origin of these accessory genes can be traced in organisms sharing a common or convergent eco-evolutionary history with the receptor (Smillie et al., 2011; Fondi et al., 2016). This asymmetrical pattern of gene transfer allows us to identify highways of gene sharing (Beiko et al., 2005).

### Antibiotic Resistance Genes in the Mobile Accessory Genome

The ensemble of antibiotic resistance genes is the resistome (D’Costa et al., 2006; Fajardo et al., 2008; Wright, 2010). The term can be applied to the resistance genes of a given bacterial population, a species or any higher taxa, or to the whole microbiota. However, the estimated size of the resistome is highly dependent on the definition of the resistance gene (Martínez et al., 2015). A mutation in a chromosomal gene might result in a resistance phenotype, but this mutated gene is rarely transferred to other bacterial species. In fact, the majority of the resistance genes detected in metagenomes are permanently associated with the same microorganisms (Fondi et al., 2016); i.e., they are intrinsic resistance genes (Olivares-Pacheco et al., 2013; Forsberg et al., 2014; Ruppé et al., 2019). However, most of antibiotic resistance genes of importance in public health are located into MGEs (Martínez et al., 2015). Any type of genetic interaction based on horizontal gene transfer favors the spread of antibiotic resistance (Huddleston, 2014). High-risk, transtaxa antibiotic resistance genes are prone to horizontal gene transfer by being included in structures such as plasmids, integrative and conjugative elements, conjugative islands, phages, and phage-like elements. To calculate the real proportion of antibiotic resistance genes among accessory genes transmitted by MGEs is presently a difficult task, given these elements are over-represented in the available databases, which are enriched with antibiotic-resistant organisms.

### Accessory Genome Interspecific Flow Channels and the Spread of Antibiotic-Resistance Genes in Gammaproteobacteria

The spread of accessory genes, antibiotic resistance genes being a fraction of these, occurs asymmetrically between bacterial species (Hu et al., 2016). The antibiotic resistance gene flow between species can be envisioned as interbacterial roads and highways, which are used by the mobile elements serving as “vessels of the communal gene pool” (Beiko et al., 2005; Norman et al., 2009). Note that antibiotic resistance function (phenotype) might depend on the horizontal co-transfer of neighbor (clustered) non-resistance genes, when are part of an operon. The operon organization is beneficial as enables the transfer of functionally coupled genes (Lawrence, 1999). Knowing the roads and highways by which the accessory genome flows should help us predict the itineraries that will be used by antibiotic resistance genes. In other words, antibiotic resistance genes circulate in the same channels as the accessory genome, comprising most genes involved in cell-environment adaptive interactions (Paquola et al., 2018).

The accessory gene flow among Gammaproteobacteria has been represented as a bipartite network, where the edges (links) connect two independent sets of entities, in our case bacterial genomes and antibiotic resistance proteins (genes) (Lanza et al., 2018; Figure 1). The distance between two bacterial species is proportional to the number of connections, that is, the number of shared proteins. This representation was based on the study of 21 Gammaproteobacterial species, represented by 47,885 genomes, analyzed using the Porous material Analysis Toolbox^4^ platform, based on AcCNET software (Lanza et al., 2017). As mentioned above, the bipartite network includes nodes belonging to different categories, in our case genomes and proteins (gene sequences were translated into proteins), so that each genome has links with their corresponding proteins. A statistical module allows inferring both genome clustering and protein clustering. Genome clustering arranges the genomes into groups (units) that share specific antibiotic resistance proteins. Protein clustering illustrates the possibility of the co-occurrence of specific proteins that are found in the same group of genomes.

This representation suggests that the accessory genome gene flow circulates in gamma proteobacteria favored by a phylogenetic neighborhood. In Enterobacterales, the flow is preferential between a “**flow core**” constituted by *Escherichia*, *Klebsiella*, *Salmonella, Citrobacter*, and *Enterobacter*, linked with the closer genera *Serratia* and *Yersinia*. Frequently transited pipelines linking this flow core to other distant species occur between *Vibrio* and *Salmonella; Escherichia-Salmonella* and *Pasteurella* and *Haemophilus*; *Klebsiella*-*Serratia* with *Acinetobacter* and *Pseudomonas.* However, many links occur outside these high roads, including a few reaching the far-located *Legionella*. With the precaution of considering the biased composition of available genetic databases, these roads correspond well with the history of recent antibiotic resistance events.

These gene flow highways are highly consistent with the genome-based phylogeny of the bacterial organisms. Seven phylogenetic groups or clades have recently been proposed in Enterobacterales (Adeolu et al., 2016). The first, the *Escherichia-Enterobacter* clade, comprises *Escherichia*, *Klebsiella*, *Enterobacter*, *Raoultella*, *Kluyvera*, *Citrobacter*, *Salmonella*, *Leclercia*, and *Cronobacter*, and corresponds to the organisms more involved in gene flow between human and animal microbiomes (Hu et al., 2016, 2017). Other clades, such as *Erwinia-Pantoea*, *Pectobacterium-Dickeya*, *Serratia-Yersinia*, *Hafnia-Edwardsiella*, *Proteus-Xenorhabdus*, and *Budvicia* can certainly be considered as candidates in transenvironmental and transmicrobiome genetic transfer of antibiotic resistance genes to the species of the *Escherichia*-*Enterobacter* clade.

## One Health Communication and the Ecology of Bacterial Species

An important corollary to the above is that by knowing the species composition and their relative frequency in a particular location, we could probably predict the local risks for communication and eventual dissemination of antibiotic resistance. Note that for such a purpose we should consider all relevant species in the various microbiotas converging in the One Health microbiosphere. However, the taxa-area relationship of bacteria, which is a critical aspect for understanding interspecies communication in One Health studies, remains difficult to establish (Horner-Devine et al., 2004). These studies should be oriented to acquire data about four main relevant issues. First, to localize the preferential or primary “reproductive sites” of the various organisms (species/clones), i.e., the natural locations where they reach the highest growth rates and population densities. Second, to identify secondary multiplication sites where they reproduce less efficiently but can reach significant population sizes. Third, to examine other environments, the “tolerated environments” where they can survive during significant periods of time, probably under very slow multiplication or persistence conditions. Fourth, to identify the “excluded environments” where these populations are unable to survive.

Connectivity of bacterial species depends on the overlapping of sites where their multiplication or persistence is possible, and thus the possibility of acquiring resistance genes or accessory genes at large. Sites where bacteria can meet and evolve resistance have been named “genetic exchange reactors” (Baquero et al., 2008). To illustrate this point, and with the awareness that this is only a partial view (excluding, for example, antibiotic gene flow in Gram-positives), in the following paragraphs we summarize the main ecological traits of the main genera of the *Escherichia*-*Enterobacter* clade that might explain transenvironmental One Health antibiotic resistance gene flow.

The genus *Escherichia*, and particularly *E. coli*, is by far the deadliest type of bacterial organism influencing human health; consequently, the control of antibiotic resistance acquisition is a critical issue (Vila et al., 2016). Most probably, the preferential reproductive site is the lower intestinal tract of warm-blooded animals. However, *E. coli* can also integrate and multiply into indigenous microbial communities in the environment (Jang et al., 2017), which might constitute secondary multiplication sites. Ecological barriers have prevented gene flow between environmental and intestinal *E. coli* lineages (Luo et al., 2011), but such hurdles are collapsing in an increasingly polluted environment. Sewage water, including water from treatment plants, allows the persistence of many related Enterobacteriaceae, predominantly *E. coli* (Vilanova et al., 2004). In addition, *E. coli* populations can persist and maintain growth potential in the soil (Byappanahalli et al., 2009). In proportion to their relative population size and replicative potential, *E. coli* can acquire resistance genes from donors at these sites. Note that most relevant antibiotic resistance genes in *E. coli* originated in environmental (non-intestinal) bacteria (Hernando-Amado et al., 2019).

*Klebsiella* is a pivotal organism in the transfer of antibiotic resistance determinants from environmental (note that *Klebsiella* is a nitrogen-fixing type of organism) to intestinal microbes. *K. pneumoniae* is ubiquitous in the environmental microbiotas surrounding humans and animals, including in water, soil, and plants. Copper-resistance is probably a good marker for soil-water versus intestinal habitat, being *Klebsiella* much more frequently resistant than the more intestinal-adapted *E. coli* (Sánchez-Valenzuela et al., 2017). There are no significant differences between environmental and clinical strains, with the possible exception of capsular antigens. Interestingly, there is a possible shift in the *K. pneumoniae* accessory genome toward human and animal adaptation (Martin and Bachman, 2018), increasing the possibility of genetic interactions with more human-animal adapted bacteria, such as *E. coli*. In fact, most of the currently threatening mechanisms of resistance, including extended-spectrum beta-lactamases (Valverde et al., 2007) and carbapenemases, as well as colistin-resistance, were introduced in the intestinal microbiota via *K. pneumoniae* (Holt et al., 2015; Rolain et al., 2016; Hadjadj et al., 2017). In fact, carbapenemase-producing *K. pneumoniae* gut colonization precedes *E. coli* acquisition of resistance (Hernández-García et al., 2019). Once undistinguished from *K. pneumoniae*, *K. variicola* has been mostly found in soil and plants (as sugar cane stems, maize shoots, and banana leaves), but has also been associated with human infections (Martínez-Romero et al., 2015). *K. quasipneumoniae* probably has an intermediate position between *K. pneumoniae* and *K. variicola* with respect to human and animal colonization. *Klebsiella oxytoca* (probably a complex genetic group of related bacteria) is now part of the consortium of environmental microorganisms that has likely contributed to the spread among human strains of antibiotic resistance, including carbapenemase genes (Khan et al., 2018), as with the related species *K. michiganensis* and *Klebsiella grimontii* (Liu et al., 2018) or the *K. huaxensis*-*K. spallanzani* group (Merla et al., 2019). However, a deeper study of the ecology of *Klebsiella* species is warranted. In general, as in the case of *E. coli*, this study should be based on the recognition of species ecotypes colonizing specific microhabitats where they can overlap with potential donors of antibiotic resistance (Koeppel et al., 2008). Each ecotype presents different opportunities for horizontal gene transfer.

Within *Enterobacter*, the *E. cloacae* complex, an environmental-animal-human genus (endophytic symbionts) that includes the cluster *E. xianfangensis* (an organism of the plant rhizosphere) and *E. hormaechei*, harbors transferable carbapenemases, suggesting an important role in resistance gene flow (Peirano et al., 2018). *E. aerogenes* is much more closely related than *E. cloacae* to the *Klebsiella* genus (*Klebsiella aerogenes*).

In the genus *Serratia*, *S. marcescens* was considered an “environment-only” organism until the 1950s and 1960s, producing pink colonies. In recent decades, many strains have been isolated from the clinical environment, all of them non-pigmented. *S. marcescens* is widespread in nature and is a frequent food colonizer, particularly in starchy foods. Strains isolated from patients are frequently antibiotic-resistant, however, many strains from the environment, including the hospital environment, are much more susceptible (Ehrenkranz et al., 1980). Resistant clinical strains are carriers of extended-spectrum beta-lactamases and carbapenemases (Yang et al., 2012).

Other environmental organisms, probably rare in the intestine, such as *Kluyvera*, in which the first CTX-M extended-spectrum beta-lactamases probably originated, might have played a key role in its early transmission to the *Klebsiella* genus and from there to *E. coli*. *Kluyvera* has been shown to belong to a resistance gene exchange community in the intestine of patients, together with *Raoultella ornithinolytica*, *K. pneumoniae*, and *E. coli* (Hernández-García et al., 2018). In fact, *Raoultella* is usually found in water and plants, but is not infrequent in human-associated isolates (Seng et al., 2016).

Finally, *Citrobacter* and *Salmonella* should also be included among shuttle species able to colonize humans, animals, plants, and the environment. High-risk transferable resistance genes, such as carbapenemases, have been consistently found in *Citrobacter* (Pepperell et al., 2002; Arana et al., 2017). The genus *Salmonella* has a known association with human and animal pathogenicity, but it also interacts with the surfaces and tissues of plants and their associated microbiota, including protists (Brandl et al., 2013).

## One Health Multilevel Dynamics of Antibiotic Resistance

**FIGURE 2:**
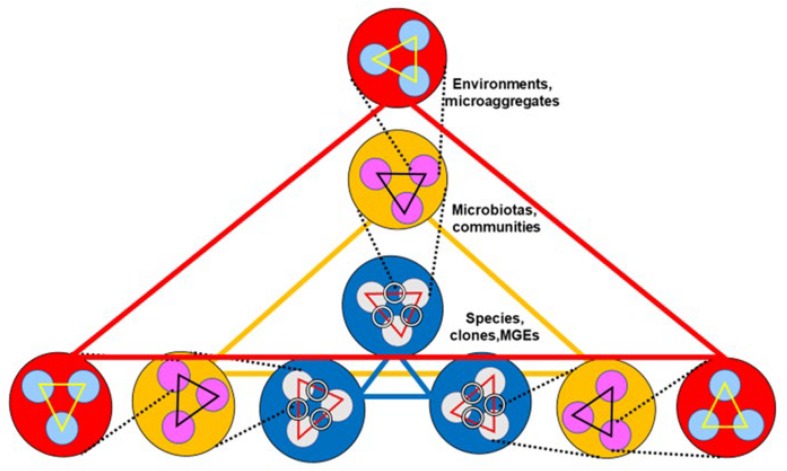
Multilevel communication between environments influencing antibiotic resistance. Communication occurs among environments (red circles and large red “communication” triangle), among the microbiotas contained in these environments (yellow circles y large yellow triangle), and among the species and clones contained in these microbiotas (blue circles and blue triangle). Inside environments there are spatially defined subenvironments or macroaggregates (light blue small circles). They contain microbiotas, bacterial community microaggregates (pink circles), which at their turn contain bacterial species and clones (light gray circles), which contain mobile genetic elements (rings, representing plasmids). At each one of these levels, communications (small triangles) are established. One Health emphasized that merging of environments, microbiotas, and bacterial communities, favors communications and consequently the spread of antibiotic resistance genes.

As presented in the preceding sections, the dynamics of antibiotic resistance is a multi-hierarchical phenomenon (Baquero et al., 2013; Hernando-Amado et al., 2019). In a highly simplified way, the first level in the One Health hierarchy (large red triangle in Figure 2) is constituted by the interactions among environments (environmental merging); typically, human, animal, plant, soil, and water environments (Thanner et al., 2016). Indeed, environmental merging occurs by gradient formation, so that a multiplicity of hybrid environments is expected to occur. In fact, such a process occurs by merging sub-environments. The matrix of many environments is composed (as it is expressed in soil ecology) by sub-environments as macroaggregates, spatially differentiated structures, containing microaggregates, typically smaller than 250 μm, composed of diverse inorganic, organic and biotic materials, where assemblies of microbial organisms (microbiota, and sub-microbiota assemblies) are located (Wilpiszeski et al., 2019). Such spatial organizations of bacterial communities and populations also occur in the lumen of the intestine (Earle et al., 2015).

The second level (large yellow triangle in Figure 2) is formed by the interactions among the microbiotas of these environments (microbiota merging), which occur by blending the microbial communities and subcommunities that compose the microbiota. The third level (large blue triangle in Figure 2) is composed of the interactions among bacterial species or clones, either of an ecological nature, such as cross nutrition, synergies, or antagonistic effects, or by being linked in genetic exchange communities (Skippington and Ragan, 2011). Genetic exchanges, including antibiotic resistance genes, are facilitated by MGEs. Of course, we can consider further levels of interaction, including the interactions among MGEs and ultimately, interactions between genes, including gene fusion or gene recombination. The modification of the conditions at each one of these levels should influence (up and down) the other hierarchical levels; for instance, the variable chemical composition of the gut lumen (the local chemosphere) influences bacterial interactions and probably microbiome merging (Baquero et al., 2019). All these interactive levels shape the emergence, spread, and maintenance of antibiotic resistance.

A problem to be addressed in research on multihierarchical systems is how to predict to which extent the changes in a given level of the hierarchy might alter the composition of the neighboring levels. This key problem in ecology, and generally in One Health, has been approached recently by computational sciences, including the application of membrane computing modeling technologies, a biologically inspired methodology that has been recently applied to the prediction of antibiotic resistance (Campos et al., 2019).

The highly integrative concept of One Health (and the highly related concept of Global Health) has provided a holistic image of the problem of antibiotic resistance, far beyond the historical consideration as a “hospital-based problem.” At the same time, the One Health approach opens the door for the investigation and development of the new biochemical, microbiological, ecological, bioinformatic, and computational tools required to understand and control the problem of antibiotic resistance on a planetary scale.

## Author Contributions

FB wrote the review. TC, J-LM, VL, and SA-G contributed with paragraphs, and provided a deep intellectual contribution of the concepts exposed.

## Conflict of Interest

The authors declare that the research was conducted in the absence of any commercial or financial relationships that could be construed as a potential conflict of interest.

## Footnotes

1 http://faostat3.fao.org/faostat-gateway/go/to/download/E/EL/E

2 https://beef2live.com/story-world-cattle-inventory-ranking-countries-0-106905

3 https://www.four-paws.us/campaigns-topics/topics/farm-animals/life-expectancy

4 https://github.com/irycisBioinfo/PATO
